# Supplementary material for: Ruthenium Decorated Polypyrrole Nanoparticles for Highly Sensitive Hydrogen Gas Sensors Using Component Ratio and Protonation Control
Source: Polymers (Basel). 2020 Jun 26;12(6):1427. doi: 10.3390/polym12061427 (PMC7361791; doi:10.3390/polym12061427)
Supplement: Supplementary file 1 [file polymers-12-01427-s001.pdf]

polymers

MDPI

Supporting information

Ruthenium Decorated Polypyrrole Nanoparticles for Highly Sensitive Hydrogen Gas Sensors Using Component Ratio and Protonation Control

Jungkyun Oh[1], Jun Seop Lee[2]*, and Jyongsik Jang[1]*

[1] School of Chemical and Biological Engineering, Seoul National University, 1 Gwanangro, Sillim-dong, Gwanak-gu, Seoul 08862, Republic of Korea

[2] Department of Materials Science and Engineering, Gachon University, 1342 Seongnam-Daero, Sujeong-Gu, Seongnam-Si, Gyeonggi-Do 13120, Republic of Korea

*Corresponding authors: jsjang@plaza.snu.ac.kr (J. Jang) and junseop@gachon.ac.kr (J. S. Lee)

1_Synthesis of Ru_CPPy

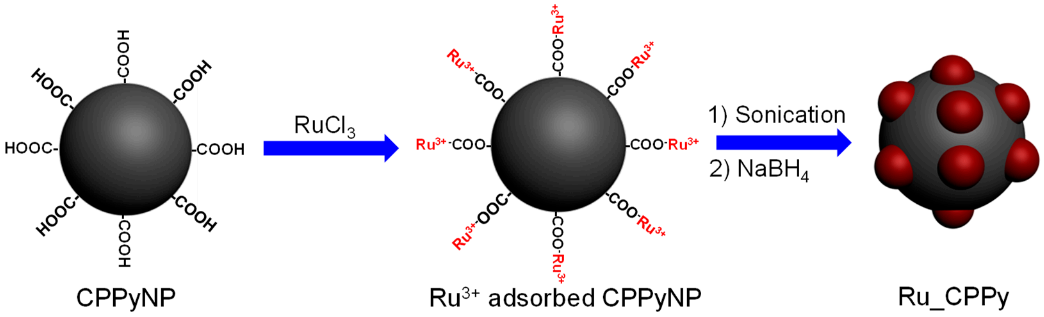

Figure S1. Schematic illustration for the fabrication process of ruthenium nanoclusters decorated carboxylated polypyrrole nanoparticles (Ru_CPPy).

2_Images of CPPyNP

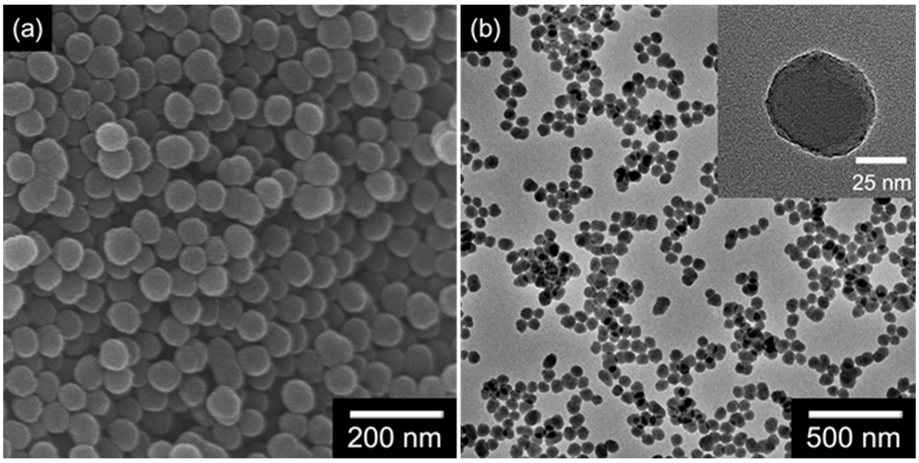

Figure S2. (a) FE-SEM and (b) TEM images of pristine CPPy NPs.

3. Ru particles without CPPyNP

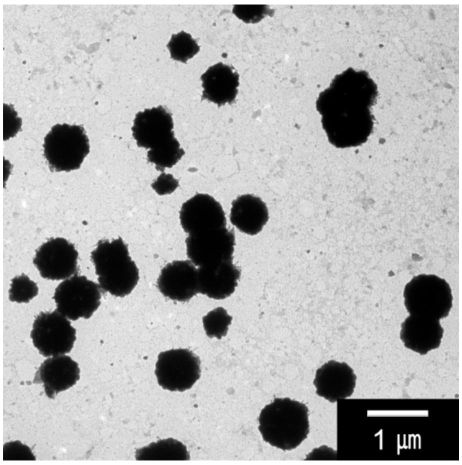

Figure S3. TEM image of Ru particles without CPPyNPs.

4_Size of Ru nanoclusters

Table S1. Average sizes of Ru nanoparticles on the surface of Ru/CPPyNPs with different concentrations of Ru precursor aqueous solution.

Material	Ru nanoparticle size (nm)
Ru_CPPy_0.5	2.0 (± 0.15)
Ru_CPPy_1.5	3.5 (± 0.20)
Ru_CPPy_3.0	6.0 (± 0.32)
Ru_CPPy_4.0	10.0 (± 4.0)
Ru_CPPy_5.0	13.1 (± 7.0)

5_XRD spectra of particles

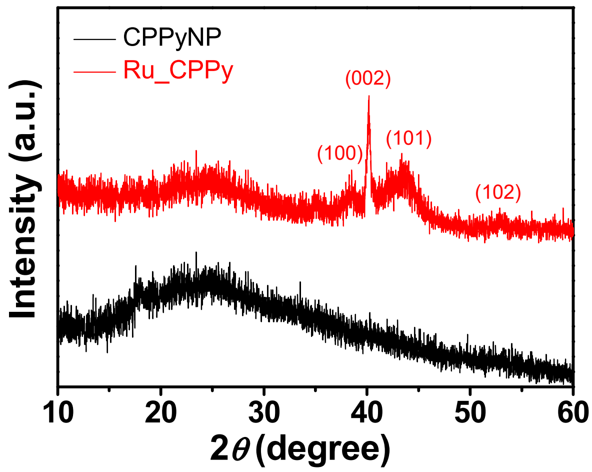

Figure S4. X-ray diffraction (XRD) patterns of CPPyNP (black) and Ru_CPPy (red).

6_Lattice structure of Ru component

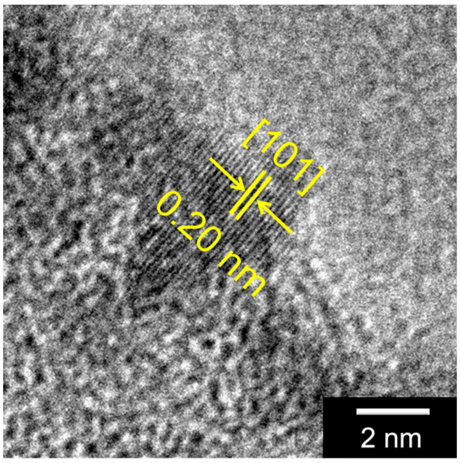

Figure S5. High-resolution transmission electron microscopy (HR-TEM) image of Ru nanocluster on the particle surface

7_ Raman and FT-IR spectra of protonated nanoparticles

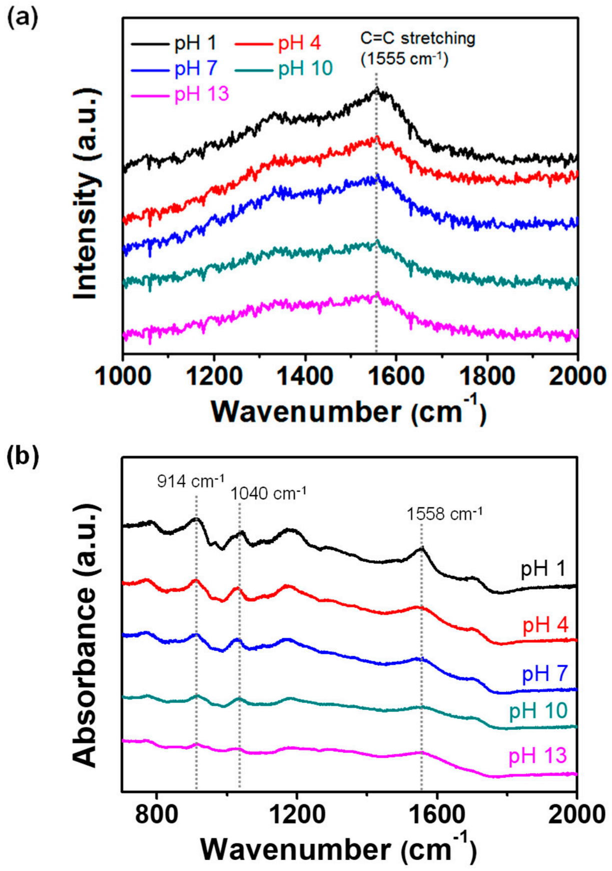

Figure S6. (a) Raman spectra and (b) Fourier-transform infrared spectroscopy (FT-IR) of Ru_CPPy with different pH treatments (black: pH 1; red: pH 4; blue: pH 7; pink: pH 10; green: pH 13).

8_ XRD spectra of particles at different pHs

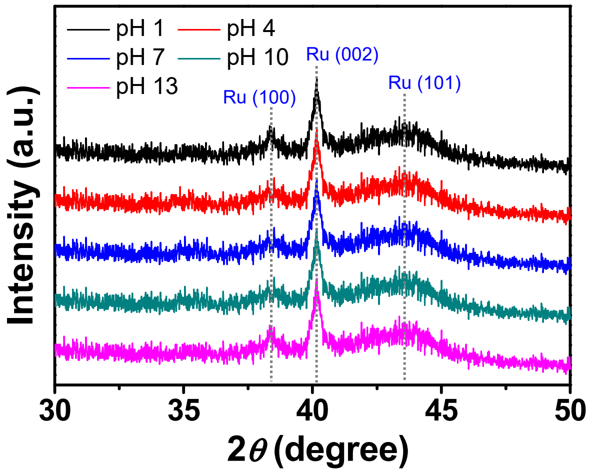

Figure S7. X-ray diffraction (XRD) of Ru_CPPy with different pH treatments (black: pH 1; red: pH 4; blue: pH 7; pink: pH 10; green: pH 13).

9_ FE-SEM images of sensor electrodes

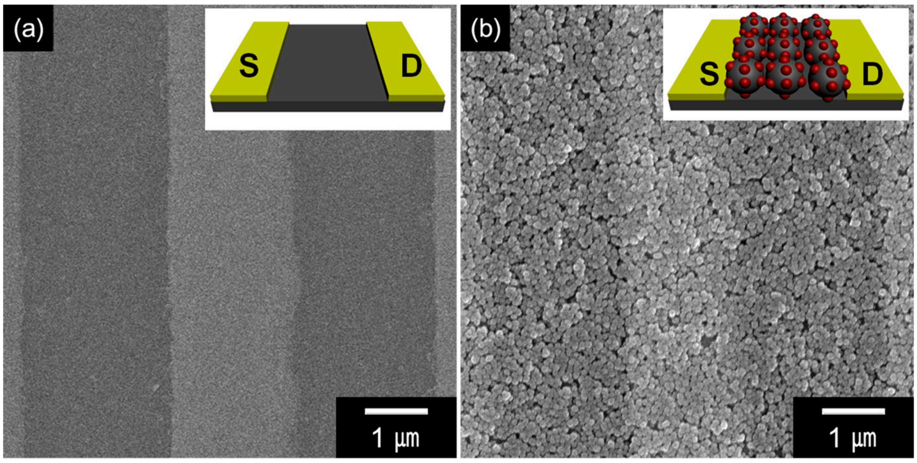

Figure S8. FE-SEM images of (a) bare interdigitated micro array (IDA) electrode and (b) Ru_CPPy decorated on the IDA substrate.

10_ Sensing performance of other nanoparticles

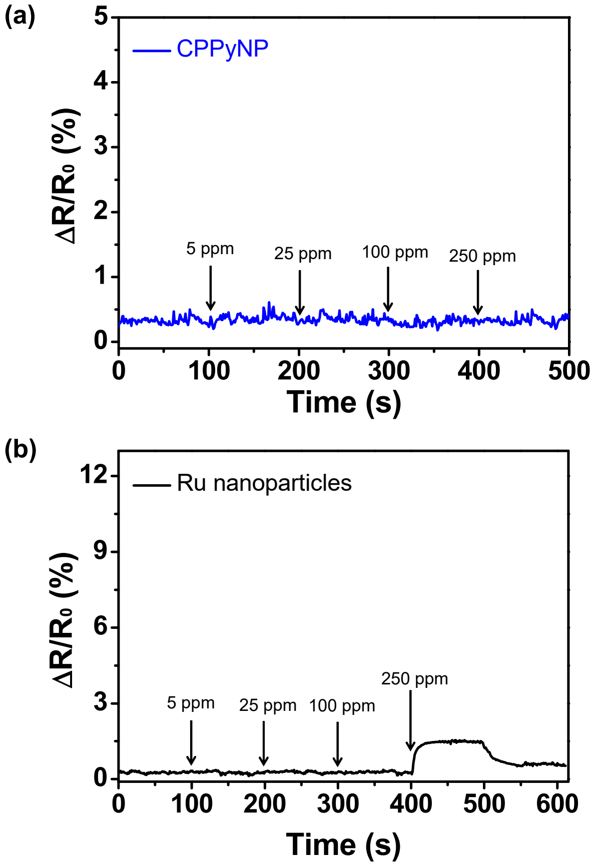

Figure S9. Normalized resistance changes upon sequential exposure to various concentrations of hydrogen gas to (a) pristine CPPyNPs and (b) Ru particles.

11_ H_2 Sensing performance comparison of electrodes

Table S2. Hydrogen gas sensing ability of different nanomaterials based sensing electrodes.

Configuration	Working temperature	MDL[a)]	Response time	Recovery time	Reference
Pd NPs[b)] on graphene	25ºC	20 ppm	≥15 min	≥30 min	[S1]
Pd NPs on graphene nanoribbons	25ºC	30 ppm	≥60 s	≥300 s	[S2]
Pd NCs[c)] on graphene	25ºC	6 ppm	20 min	54 min	[S3]
Pd-NiO particle	150ºC	30 ppm	131 s	151 s	[S4]
Ru_CPPy_3.0	25ºC	0.5 ppm	31 s	58 s	This work

[a)] Minimum detectable level, [b)] nanoparticles, [c)] nanocubes

12_ Sensing performance with temperature difference

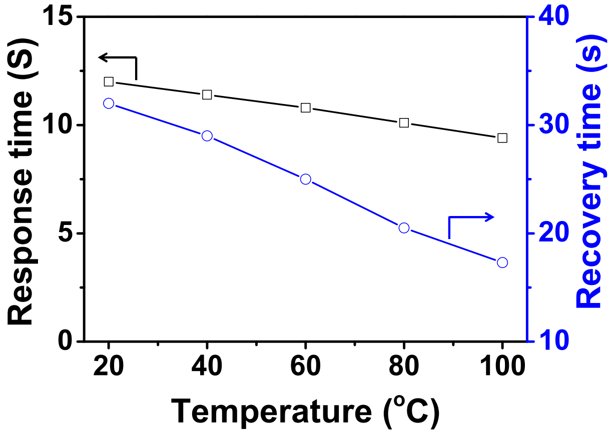

Figure S10. Response and recovery time changes of the pH 1 electrode with working temperature variation.

13_ Morphology images of nanoparticles before and after H_2 detection

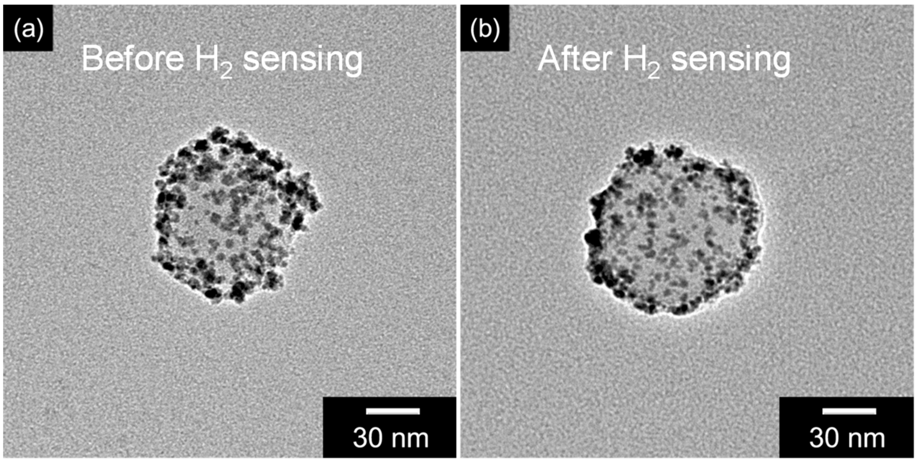

Figure S11. Transmission electron microscopy (TEM) images of Ru_CPPy (a) before and (b) after hydrogen sensing.

14_ Selectivity of the sensor electrode

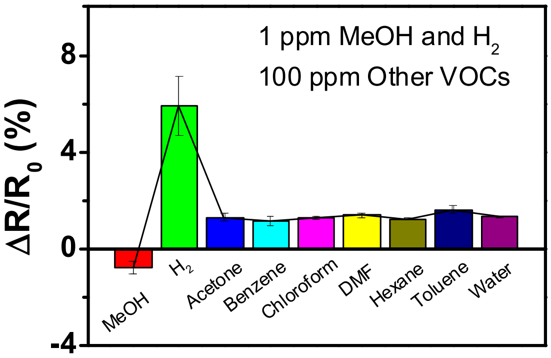

Figure S12. Normalized resistance changes of the electrode do different analytes.
